# Facial nerve stimulation in normal pigs and healthy human volunteers: transitional development of a medical device for the emergency treatment of ischemic stroke

**DOI:** 10.1186/s12967-018-1398-6

**Published:** 2018-02-15

**Authors:** Olivia Sanchez, Andrea García, Fernando Castro-Prado, Miriam Perez, Rafael Lara-Estrada, Martin Ramirez-Meza, Montserrat Godinez, Michael L. Coco, Joaquín Azpiroz, Mark K. Borsody, Emilio Sacristán

**Affiliations:** 1 National Center for Medical Imaging and Instrumentation Research, Universidad Autónoma Metropolitana-Iztapalapa, Mexico City, Mexico; 2 NeuroSpring, 8 The Green, Dover, DE 19901 USA; 3 Nervive Inc., 526 S. Main St. Suite 801-A, Akron, OH 44311 USA; 4 Central Norte Pemex, Hospital Angeles Pedregal, Mexico City, Mexico

**Keywords:** Magnetic stimulation, Facial nerve, Cerebral blood flow, Ischemic stroke

## Abstract

**Background:**

Magnetic stimulation of the facial nerve has been tested in preclinical studies as a new, non-invasive emergency treatment of ischemic stroke that acts by increasing cerebral blood flow (CBF). The objective of the studies reported herein was to identify minimal stimulation parameters that increase CBF in large animals and then test those stimulation parameters in healthy volunteers for safety, tolerability, and effectiveness at increasing CBF. This translational research is necessary preparation for clinical studies in ischemic stroke patients.

**Methods:**

Initial experiments in anesthetized Yorkshire pigs were undertaken in order to identify the lowest stimulus power and duration that increase CBF. A full 3 × 3 factorial design was used to evaluate magnetic stimulation of the facial nerve at various stimulation powers (1.3, 1.6, and 1.9 Tesla field strength at coil surface) and for various durations (2, 3.5, and 5 min). CBF was measured with contrast MRI perfusion imaging and the internal carotid arteries were assessed with MR angiography. Magnetic facial nerve stimulation with parameters identified in the pig study was then applied to 35 healthy volunteers. Safety was assessed with adverse event reports and by medical examination. Tolerability was defined as each volunteer’s ability to withstand at least 2 min of stimulation. Volunteers could determine the maximum power of stimulation they received during a ramp-up period.

**Results:**

In pigs, unilateral facial nerve stimulation increased CBF by as much as 77% over pre-stimulation baseline when administered across a range of 1.3–1.9 Tesla power and for 2- to 5-min duration. No clear dose–response relationship could be observed across this range, but lower powers and durations than these were markedly less effective. The effect of a single stimulation lasted 90 min. A second stimulation delivered 100 min after the first stimulation sustained the increased CBF without evidence of tachyphylaxis. In human, bilateral facial nerve stimulation caused only non-serious adverse events that were limited to the 2-min stimulation period. Tolerability was greatly improved by gentle encouragement from the study staff, which enabled most volunteers to tolerate 1.6–1.8 Tesla of stimulation power. CBF measures taken approximately 10 min after stimulation demonstrated on average a 32 ± 6% increase in CBF, with ≥ 25% increases in CBF occurring in 10 of the 31 volunteers who had adequate CBF measurements.

**Conclusions:**

The minimal effective stimulation parameters defined by increased CBF, as identified in the pig study, translated into safe, tolerable, and effective stimulation of healthy volunteers. These results support the future development and evaluation of non-invasive facial nerve stimulation for the emergency treatment of ischemic stroke.

*Trial Registration* retrospectively registered with clinicaltrials.gov NRV_P1_01_15 on June 6, 2017

## Background

Stroke is the leading cause of severe disability and the second leading cause of death worldwide. Ischemic stroke—which is the majority of all strokes—is caused by the occlusion of a cerebral artery, typically with a blood clot. The occlusive blood clot causes a critical loss of cerebral blood flow (CBF) to a brain region and thus death of the affected brain tissue. Emergency treatment for ischemic stroke is available in the form of intravenous tissue plasminogen activator (rtPA) and endovascular clot retrieval catheterizations, which either enzymatically dissolve the occlusive blood clot or physically remove it, restoring CBF. But these standard-of-care treatments are rarely used because of the need for specialized personnel and the numerous contraindications to treatment.

Dilation of the cerebral arteries is a well-known effect of facial nerve stimulation that increases CBF and reverses the effects of ischemic stroke in animal models [1–10]. Indeed, one company (BrainsGate) is in late-stage clinical testing of an invasive facial nerve stimulator as a treatment for ischemic stroke in the 8–24 h post-stroke therapeutic window. In contrast, our research team is developing a non-invasive magnetic facial nerve stimulator for clinical use. Our device—called the VitalFlow™ stimulator—places proprietary magnetic stimulation coils on both sides of the head so that the magnetic field is focused upon the geniculate ganglion region of the facial nerve. The axis of the ear canal is oriented at the geniculate ganglion region of the facial nerve, which is the last portion of the nerve to contain the autonomic fibers that at that point separate from the facial nerve trunk as the petrosal projections to the cerebral arteries.

In clinical use at specialized “Stroke Center” hospitals, the VitalFlow stimulator could improve delivery of intravenous rtPA to the site of the occlusive blood clot and allow easier navigation of endovascular catheters to retrieve the occlusive blood clot. The VitalFlow could also provide rtPA- and endovascular catheter-ineligible patients an emergency treatment option. At non-Stroke Center hospitals, VitalFlow treatment would be administered to ischemic stroke patients prior to transport to a Stroke Center for definitive treatment, thereby expanding the availability of stroke healthcare services and reducing the time from stroke onset to an initial brain-saving treatment.

Herein we report the results of translational research with the VitalFlow stimulator. We first describe normal pig experiments that defined the relationship between select stimulation parameters and the CBF response for the purpose of estimating stimulation parameters that can be be used in human testing. We then report the first-in-man test of a clinical prototype VitalFlow stimulator: a study demonstrating the safety, tolerability, and effectiveness of increasing CBF by VitalFlow stimulation in healthy volunteers.

## Methods

### Study in normal pigs

#### Animal subjects

Twelve adult Yorkshire pigs between 15 and 35 kg were used in these experiments. Three stimulation trials were performed in each pig, allowing 7 days between each stimulation trial to ensure full recovery. The Institutional Animal Care and Use Committee (IACUC) of the Universidad Autónoma Metropolitana Iztapalapa approved the protocol for pig experiments to be conducted at the National Center for Investigation of Medical Instrumentation and Imaging (CI3M) laboratory. The study was carried out in accordance with the recommendations of the Guide for the Care and Use of Laboratory Animals [11], per university policy, and were reported in compliance with the Animal Research Reporting In Vivo Experiments (ARRIVE) guideline.

Pigs were housed in individual cages at a breeding facility that is specialized and licensed for research animals. Prior to, and between, experiments, the pigs were allowed access to food and water *ad libitium*. On the day of the experiment, the pig was transported to the CI3M laboratory and within 3 h of arrival was anesthetized using intramuscular azaperone (2 mg/kg) and ketamine (15 mg/kg). Isoflurane (1–2%) in 100% oxygen (3.2 L/min) was used for the maintenance of anesthesia. The pig was intubated after induction for mechanical ventilation and the maintenance of the anesthesia [12, 13], but the animal was not paralyzed.

We did not measure arterial blood gas samples because the repeated evaluation of the pigs over multiple weeks in this manner would have created hemorrhage and infection risk. The experimental set-up including the use of pure (100%) oxygen for ventilation is routinely used in the CI3M laboratory, and doing so maintains sustainable blood gas measures for several hours even in physiologically-unstable experimental models [14–16]. Pure oxygen was required because: (1) the experiments were conducted in Mexico City, where the high altitude lowers the effective partial pressure of the pure oxygen by about 77% to 1.3 ATM; and (2) added lengths of ventilation tubing were necessary for the pig to fit into the MR scanner, which created dead space in the ventilation system. Use of 1.3 ATM oxygen for brief periods of time such as the ≈ 5 h-long experiments we report here, does not approach the threshold for either lung or brain toxicity [17] and instead may reduce post-procedural hypoxia and infections [18].

**Fig. 1:**
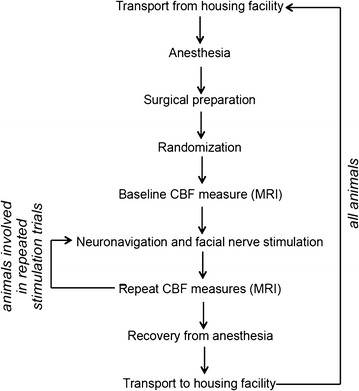
Schematic of the pig study procedures. Pigs subject to repeated stimulation trials in a single experiment required repeated neuronavigation positioning of the stimulation coils and facial nerve stimulation after measurement of the cerebral blood flow response to an initial stimulation. All pigs were subject to multiple experiments separated by a 7-day recovery period at the animal housing facility

The lateral auricular vein was catheterized for contrast injection and for venous blood gas sampling. Throughout the experiment, the pig was secured to a board with straps, allowing for easy movement of the pig by the study team and for reproducible placement of the pig in the MRI scanner. Anesthetization and surgical preparation took about 30 min to complete, and the animal progressed immediately into the experimental procedures shown in Fig. 1. After experimental use, the isoflurane was discontinued and the pig was extubated under the direction of the study veterinarian once it began coughing. The pig was recovered on-site and then transported back to the animal facility for housing between experiments.

#### Neuronavigation and facial nerve stimulation

Neuronavigation-guided positioning of the stimulation coil involved a reference system based on fiducial markers glued symmetrically around the pig’s head. Benzonate gelcaps (100 mg) were used as fiducial markers. Neuronavigation-guided positioning of the stimulation coil was performed as described previously [19, 20] using a commercially-available neuronavigation system (Brain Science Tools; Utrecht, the Netherlands). Briefly, the target segment of the facial nerve (the intracanalicular segment at the point of the geniculate ganglion) was identified by its location anterior to the semicircular canals, lateral and inferior to the cochlea, and medial to the ear canal. T2-weighted MRI images reconstructed on MRIcron software allowed for target localization based on the surrounding anatomical features.

A commercially-available 6.5 cm figure-8 magnetic stimulation coil (Cool B65; MagVenture; Copenhagen, Denmark) cooled by a circulating fluid pump and powered by a stimulus generator (MagPro R30) was used in the pig experiments [19, 20]. The stimulation coil was placed over the left ear of the pig and held in place using a mechanical arm. The manufacturer’s specifications of the stimulator coil and stimulus generator indicate that 100% power output creates a magnetic field of 2.0 Tesla strength at the surface of the stimulation coil. Stimulation in all experiments involved 280 μsec biphasic pulses delivered continuously at 10 Hz.

Stimulation power and duration were selected based on our previous experiments using sheep and dogs with the aim to find the minimal parameters that increased CBF [19]. In our previous study, stimulation at 1.0 Tesla power was found to be ineffective at increasing CBF, whereas 1.5 Tesla power was found to reliably increase CBF. Accordingly, in the current experiments, we initially set the lowest tested power at 1.3 Tesla power, whereas an intermediate stimulation power was set at 1.6 Tesla and the highest power was set at 1.9 Tesla. Similarly, the previously-tested stimulation duration was arbitrarily chosen as 5 min [19], so herein we evaluated stimulation durations of 2, 3.5, and 5 min. Based on the results of those initial experiments, an additional group of pigs was added to test the effect of a low level of stimulation (1.0 Tesla power at 1 min) and compared with a sham level of stimulation (0.1 Tesla power at 30 s).

#### Image Acquisition and Processing

MRI employed a Philips^®^ Achieva 3T scanner and an 8-channel SENSE^®^ head coil. T1- and T2-weighted images of the pig’s head and neck were acquired and used as reference for location and position of the stimulation coil, as described above. Transfer of the pig from the experimental suite where surgical preparation and facial nerve stimulation were performed to the MRI scanner was a well-practiced procedure involving a team of six that took less than 2 min to perform. No changes in the ventilator equipment, including tubing length or ventilator settings, were involved in the transfer.

**Fig. 2:**
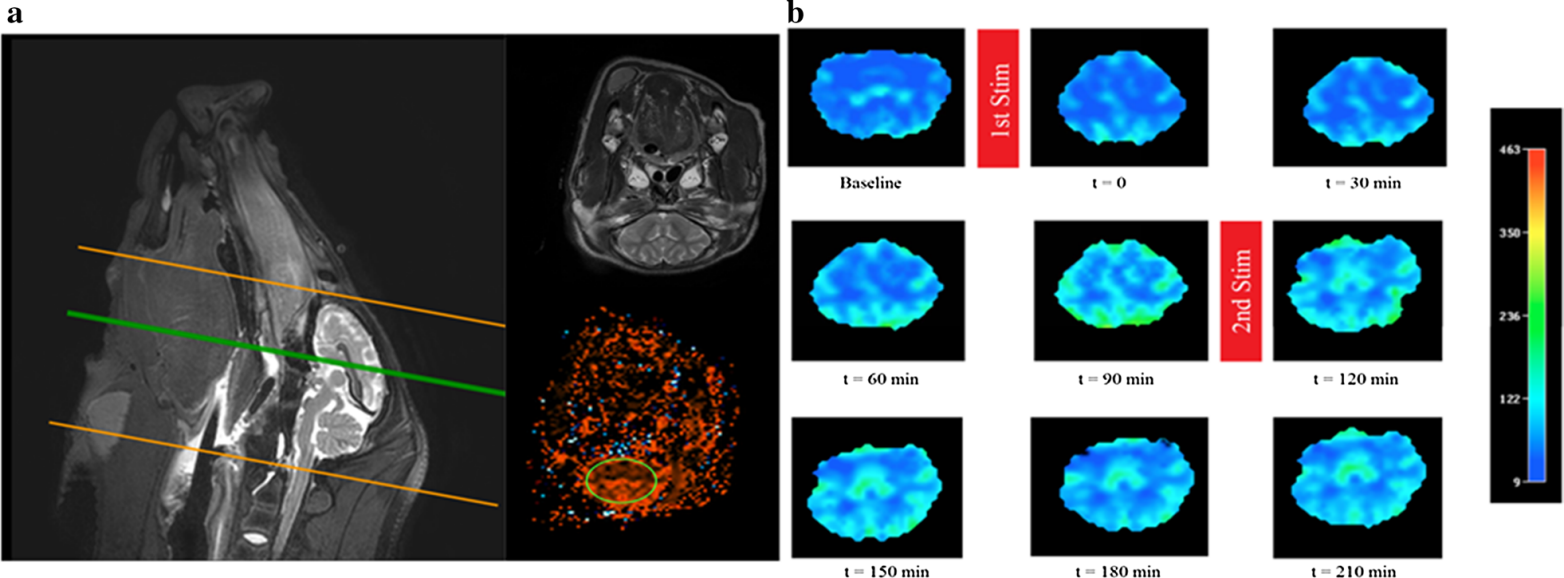
An example of the cerebral blood flow response to magnetic facial nerve stimulation in the pig. **a** MRI T2 weighted image used as an anatomical reference (top) for ROI placement (bottom). **b** MRI perfusion images ordered temporally from left-to-right and top-to-bottom, with the intervening red bars demonstrating when the first and second stimulations were delivered. The example is a pig subject to repeated facial nerve stimulation with 1.9 Tesla power for 5 min. The oval ROI shown in **a** was created with Osirix software for quantification purposes; the manual segmentation shown in **b** was created with Philips software for graphical representation only

Perfusion MRI involved contrast agent Gadovist^®^ 1.0 mmol. (0.1 mL/kg followed by a 15 mL saline flush) and PRESTO Philips^®^ sequences. Each MRI assessment obtained 25 perfusion maps of the brain. To keep the same region of interest (ROI) area, five central maps were used for analysis corresponding to slices 11–15. Slices 11–15 are centrally located along the rostral-caudal axis of the brain and thus have the maximum cross-section area of brain tissue inside the ROI (Fig. 2a). For calculation of perfusion index measures of CBF, brain tissue segmentation was performed by the Osirix software using an oval region of interest (ROI) with a 12 cm^2^ area for each analyzed slice. Perfusion index was calculated based on the area-under-the-curve of the first pass of contrast agent. The mean values were then exported and normalized against the respective pre-stimulation baseline value for each pig.

MRI angiography of the internal carotid arteries was employed to quantify any arterial dilation. Using Osirix Software, five slices were selected distal to the bifurcation of the common carotid arteries. Two dimensional ROIs were located on each slice by placing the cursor in the center of each internal carotid artery in cross-section and then expanding the ROI using the region-growing algorithm. Arterial cross-section areas were averaged across the five slices and normalized against the pre-stimulation baseline cross-section area for that artery.

#### Experimental designs

##### Experiments involving repeated stimulation trials

The initial design of the pig experiments was a repeated stimulation trial design. The first nine pigs were subject to repeated stimulation trials in which two stimulations were delivered during the same experiment in series. The first stimulation was delivered as described above. Then, after the 90 min MRI assessment, a second stimulation using the same parameters was delivered; the second stimulation occurred about 100 min after the first stimulation. Thereafter, the animal was returned to the MRI scanner so as to keep the original schedule for imaging at 30 min intervals, and another 90 min of monitoring with MRI at 30 min intervals was obtained prior to recovering the pig.

A full factorial experiment design was used in the repeated stimulation experiments, employing two variables at three levels. Thus, three stimulation durations (2, 3.5, and 5 min) and three power parameters (1.3, 1.6, and 1.9 Tesla power) were assessed in nine possible combinations. Forced allocation randomization was employed to ensure equal group sizes.

##### Experiments involving a single stimulation trial

After completing the repeated stimulation trial experiments described above, it became apparent that all combinations of the stimulation parameters selected were comparably effective at increasing CBF and causing cerebral artery dilation. In other words, the CBF and cerebral artery caliber measurements provided by the repeated stimulation trial experiments appeared to be on the plateau of a dose–response curve. In order to assess the lower end of that dose–response curve, we subsequently added an additional set of experiments using lower stimulation powers and times, as described below.

Single stimulation trials were performed in three pigs on three consecutive weeks (i.e., one stimulation per week). The parameters used in each of the three pigs were: 1.9 Tesla power for 2 min; 1.0 Tesla power for 1 min (“low stim”); and 0.1 Tesla power for 30 s (“sham stim”). We considered the sham stimulation condition to be the control condition for the experiment, since the minimal stimulation pulse power does not even trigger cutaneous sensation in skin pressed against the stimulation coil. After anesthetization and surgical preparation, an MRI study was performed to support neuronavigation and also to obtain a baseline perfusion index and angiography measures. After placement of the stimulation coil, which was completed within 10 min of the pre-stimulation baseline MRI, the pig was stimulated with one of the three sets of parameters selected in a random manner so as to ensure each pig was ultimately subjected to all three sets of stimulation parameters (forced allocation randomization). Immediately after stimulation, the pig was returned to the MRI scanner for post-stimulation imaging. Post-stimulation imaging was collected at 30 min intervals from the immediate post-stimulation (t = 0) time point to 240 min post-stimulation, after which the animal was recovered and returned to its housing facility.

#### Statistics

The sample size was calculated a priori based on the assumption of a 40% increase in CBF over baseline caused by stimulation, an inter-individual variability of 20%, beta = 0.8 (i.e., power = 80%), and alpha = 0.05 (one-tailed). Data are expressed as the change in CBF using the animal’s own baseline CBF. Within-group data were analyzed by paired t test corrected by the Bonferonni method for multiple comparisons. Analyses involving multiple groups subject to different stimulation power or duration were made using ANOVA. Timeline comparisons were made with repeated-measures ANOVA.

### Study in healthy volunteers

#### Healthy volunteers

The Medica Sur Ethics Committee and the Metropolitan University Ethics Committee approved the human volunteer study, which was conducted under Good Clinical Practices (GCP) with auditing. Informed consent was obtained from all volunteers. The study was carried out in two parts: Part 1 assessed a full panel of safety measures including aural and ophthalmologic tests in addition to tolerability and CBF responses; Part 2 discontinued aural and ophthalmologic testing based on the results of Part 1, but it continued to assess other measures of safety, tolerability, and CBF changes.

In Part 1, a total of 24 people (13 men and 11 women) were enrolled. To be eligible for the study, volunteers had to have no medical conditions either active or in the past. As a condition of enrollment, volunteers also had to have normal aural, otologic, and ophthalmic examinations as determined by a contracted clinical audiologist and ophthalmologist. Similarly, prior to stimulation, volunteers had to have normal brain MRI, MR angiography, and neurological examinations as determined by a neurosurgeon (F. Castro-Prado). All volunteers were between 20–40 years-of-age and the average age of the group was 23.6 years.

In Part 2, an additional 13 volunteers were enrolled, bringing the total group size to 37 people (30 men and 17 women). To be eligible for Part 2 of the study, volunteers had to be free of renal, neurological, or cardiovascular disease, and had to have normal brain MRI, MR angiography, and neurological examination as determined by the neurosurgeon. All volunteers were between 20–40 years-of-age, and the additional volunteers changed the overall average of the group to 23.9 years.

After enrollment, two volunteers from Part 1, both males, had to be excluded on the day of stimulation when they revealed that they had preexisting medical conditions. This left 35 people (28 men and 17 women) total in the study.

#### Neuronavigation and facial nerve stimulation

**Fig. 3:**
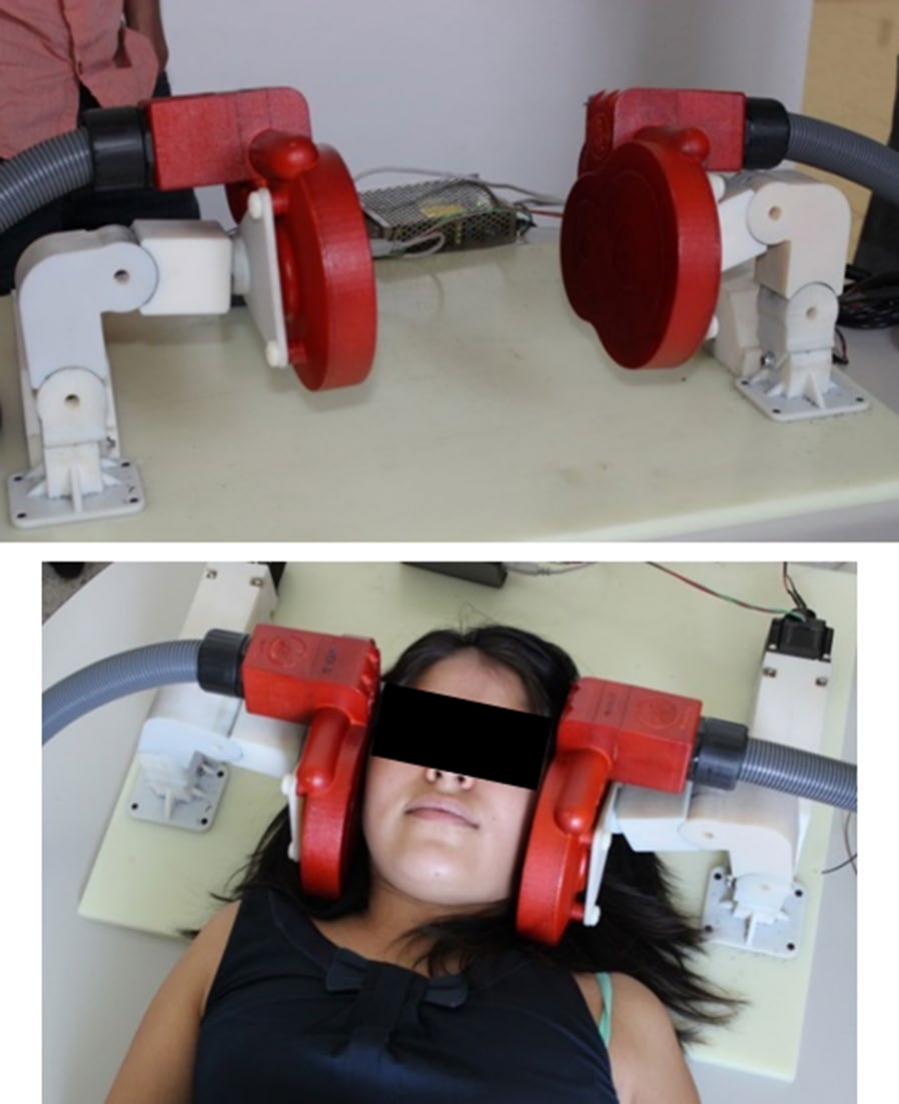
The clinical prototype VitalFlow stimulator headrest and stimulation coils used in the healthy volunteer study. Stimulus generator and cooling system components not shown

A clinical prototype VitalFlow stimulator was designed for use in humans (Fig. 3). Specific attention was given to the stimulation coil structure, which was customized for stimulation of human head anatomy. The stimulation coil designed for the human head reduces brain exposure to magnetic energy by 70% according to computer modeling research [21] while maintaining comparable induced electrical field strength at the facial nerve target. Stimulation power thus should translate directly from pig to human, since the depth from the coil surface to the facial nerve target is comparable between the species [3.8 ± 0.4 cm in pig (n = 8) versus 4.2 ± 0.1 cm in man (n = 5); mean ± STD]. The stimulation coils were mated to stimulus generators and circulating fluid cooling systems (Neurosoft; Ivanovo, Russia), and the two functional magnetic stimulators were yoked together to provide synchronized, bilateral stimulation.

For all volunteers, neuronavigation was performed based on T1 and T2 MRI reconstructions and the geniculate ganglion was identified bilaterally as described in the pig study. After the adequate placement of the stimulation coils in a fixed position made possible by a headrest with lockable arms, stimulation was delivered in biphasic pulses of 280 μs at 10 Hz, which we previously demonstrated to be more effective than other stimulation frequencies in animal testing [19, 20].

The selection of stimulation parameters for the human study was driven by the ethical aim to deliver the least amount of stimulation needed to increase CBF. Stimulation with the least amount of power and for the shortest duration also should maximize safety and tolerability, and it would minimize power demands and heating of the device. However, we also recognized the different tolerance that individuals in the healthy volunteer study would have for the stimulation. Accordingly, the power of the magnetic stimulation was adjusted to each volunteer’s individual level of toleration in a stepwise fashion. Stimulation started at 0.8 Tesla power for 10 s, and only after the volunteer indicated ‘thumbs up’ approval was the power increased by 0.2 Tesla for another 10 s period. Then, the volunteer could decide in favor of another increase in stimulation power with a ‘thumbs up’ signal or disapprove of the last increase with a ‘thumbs down’ signal, which led to a 0.2 Tesla reduction in stimulation power. When the volunteer decided to maintain stimulation at a certain power level, he or she shook the hand side-to-side, at which point the stimulation was continued at that power for a period of 3 min. After the first 8 volunteers were stimulated, it became apparent that the volunteers could be encouraged to attempt higher stimulation powers, and so gentle encouragement was thereafter given by the study investigator (A. Garcia) during the ramp-up phase of the experiment.

#### Safety and tolerability

The study procedures are shown in Table 1. The volunteers enrolled in Part 1 of the study had ophthalmologic (intraocular pressure) and aural (audiometric graph, stapedial reflex, and Frenzel maneuvers) evaluations before and 24 h after stimulation. All volunteers had a neurological examination (cranial nerves, sensation, reflexes, motor strength) prior to and 24 h after stimulation. Adverse events were spontaneously reported by volunteers throughout the study. Adverse events of interest were also queried immediately after stimulation by the study investigator according to Table 2; the adverse events of interest were expected as a results of stimulation based on the neuroanatomy of the facial nerve and nearby inner ear structures. Tolerability was defined as the stimulation power a volunteer could receive continuously for at least 2 min, with 1 min of increasingly-powerful stimulation (ramp-up) preceding.

**Table 1:** Procedure visits for the healthy volunteer study

Procedure	Visit 1: eligibility	Visit 2: stimulation	Visit 3: 24 h post-stimulation
Informed consent	X		
Eligibility criteria review	X		
Medical evaluation	X		X
MRI baseline		X	
MRI post-stimulation		X	
Intraocular pressure	X		X
Aural evaluation	X		X
Neurological evaluation pre- and post-stimulation		X	
Stimulation		X	
Adverse event reporting	X	X	X
Adverse event query		X	
Concomitant medications	X	X	X
Device events	X	X	X

**Table 2:** Queried adverse events after stimulation

Did you feel vertigo or the sensation of movement?
Did you sense visual flashes?
Did you feel pain in stimulation area?
Did you feel pain in another area?
Did you have ringing in the ears?
Did you feel nauseated?
Did you have an abnormal taste sensation?

#### Efficacy

Efficacy of stimulation was evaluated as the change in perfusion index measures of CBF by contrast-enhanced MRI. The change in perfusion index between pre-stimulation baseline and post-stimulation was measured for each volunteer. Post-stimulation MRI was initiated approximately 10 min after stimulation ended because of the time required to position the volunteer in the MR scanner. Previously, we determined that the intra-individual variability of the perfusion index measure of CBF in our MR scanner is ± 25%. Therefore, any change in perfusion index that was < 25% was considered as a “non-responder” in this study.

Five volunteers did not provide usable CBF data. Technical issues with the MRI scanner rendered the data unusable in four volunteers and a fifth volunteer did not receive a complete stimulation due to overheating of the VitalFlow, the consequence of an incomplete cooling line purge.

#### Image acquisition

MRI was performed on a 3T MRI scanner (Achieva; Philips Healthcare), using an eight channel brain coil. We used T1W_3D_TFE (TR/TE = 7.5/3.4 ms, flip angle = 8°, FOV = 250 mm), a T2 W (TR/TE = 2500/390 ms, flip angle = 90°, FOV = 250 mm), a PRESTO (TR/TE = 17/25 ms, flip angle = 7°, FOV = 230 mm, Nr of Dynamics = 50, Dummy 5), MIP 3D_PCA (TR/TE = 25.6/3.5 ms, flip angle = 18°, FOV = 220 mm), ASL (TR/TE = 4000/10 ms, flip angle = 90°, FOV = 240 mm, Nr of Dynamics = 50), 3D_PCA (TR/TE = 16.2/4/4 ms, flip angle = 7°, FOV = 150 mm).

#### Image processing

The perfusion index maps were generated with the Philips software, using the INDEX maps we selected. Specifically, from 30 slices, we selected slices 12–23. The brain was identified in these slices and for each slice we drew an oval-shaped ROI. Then, within each ROI we obtained the mean and the standard deviation of the perfusion index. The ROI was propagated in all the selected slices taking care that only brain tissue was processed.

Perfusion analysis per group was done with ImageJ 1.45 s developed by the National Institutes of Health, USA. Dicom Image was loaded and a ROI with a 153,355 area applied to each slice using multi-measure plug-in.

#### Data analysis

Safety data was analyzed on an individual basis and according to the distribution across stimulation powers. CBF responses were reported as the change in perfusion index between pre-stimulation baseline and post-stimulation measures. Individual volunteer data (the average ± SEM of measures across the slices) was analyzed by linear regression against the stimulation power received by the volunteers.

## Results

### Study in normal pigs

As judged by routine veterinary monitoring, no pig was found to have a neurological or behavioral abnormality after magnetic facial nerve stimulation—even after as many as six cumulative stimulations. Furthermore, magnetic facial nerve stimulation did not affect vital signs during the experiments (data not shown), confirming previous reports [5, 7, 8, 19].

**Fig. 4:**
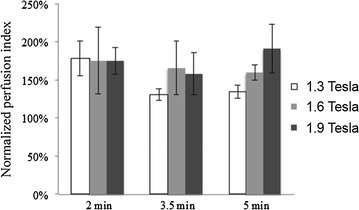
Cerebral blood flow responses to various combinations of magnetic facial nerve stimulation parameters in pigs—analysis of repeated stimulation experiments only. Normalized perfusion index of cerebral blood flow. Mean ± SE, n = 3 per group. ANOVA showed no significant difference across the various combinations of power and duration (P > 0.70)

**Fig. 5:**
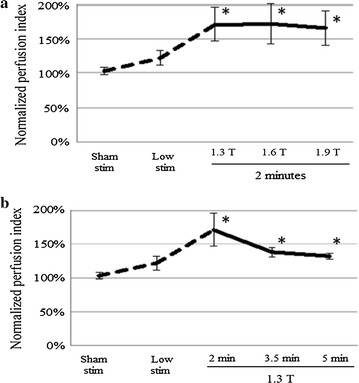
Cerebral blood flow responses to various combinations of magnetic facial nerve stimulation parameters in pigs—analysis including single stimulation experiments. **a** Effect of stimulation power; **b** effect of stimulation duration. First stimulation trial of the repeated stimulation experiments shown (n = 9 per group). Single stimulation experiments with sham stimulation (0.1 Tesla power for 30 s) or low stimulation (1.0 Tesla power for 1 min) parameters (n = 3 per group) included as dashed lines. Data from single stimulation and repeated stimulation trials are shown together to estimate the lower end of the dose–response curve, which is not seen when evaluating only the repeated stimulation trials as per Fig. 4. *P < 0.05 versus sham stim, paired t test corrected by the Bonferroni method for multiple comparisons. Mean ± SE

Stimulation with powers from 1.3 to 1.9 Tesla and durations from 2 to 5 min increased CBF in the majority of trials for about 90 min (Fig. 2). The increase in CBF occurred throughout the brain without obvious preference for the hemisphere ipsilateral to stimulation. Comparison of the CBF response to stimulation with different combinations of stimulation power and stimulation duration is shown in Fig. 4. ANOVA using only the data from the repeated stimulation trials (full factorial experimental design) showed no significant difference across the various combinations of power and duration, with all combinations increasing CBF to comparable degrees over pre-stimulation baseline. This observation led us to theorize that the assessed stimulation parameters were on the plateau of a dose–response curve, and thus we conducted additional experiments (single stimulation trials) to assess the lower end of the dose–response curve. When examined as dose–response curves that included sham stimulation (0.1 Tesla power for 30 s) and low stimulation (1.0 Tesla power for 1 min) trials, a sharply-rising dose–response curve became evident with a plateau achieved at stimulation with 1.3 Tesla power for 2 min (Fig. 5). An effect was observable at 1.0 Tesla power for 1 min, but was much more robust at higher stimulation powers and durations (P < 0.03).

With stimulation powers ≥ 1.3 Tesla power and durations ≥ 2 min, CBF increased in the range of 30-90% above the pre-stimulation baseline in most stimulation trials. On average, the CBF increased by 77% over baseline. However, it is important to note that the group averages include stimulation trials that, per our previous criteria, would have been considered as non-responsive (i.e., CBF increase < 25%). Overall, four stimulation trials had CBF responses < 25% at maximum:

- One stimulation trial of 1.3 Tesla power for 3.5 min—CBF maximal increase of 15% after a second stimulation trial;
- Two stimulation trials of 1.6 Tesla power for 2 min—CBF maximal increase of 15% after a first stimulation trial and 18% after a second stimulation trial (different pigs);
- One stimulation trial of 1.9 Tesla power for 2 min—CBF maximal increase 6% after a first stimulation trial.

Performing the ANOVA comparing the various combinations of stimulation power and duration but excluding the aforementioned four stimulation trials did not affect the results. The non-responding trials were included in all data analyses.

**Fig. 6:**
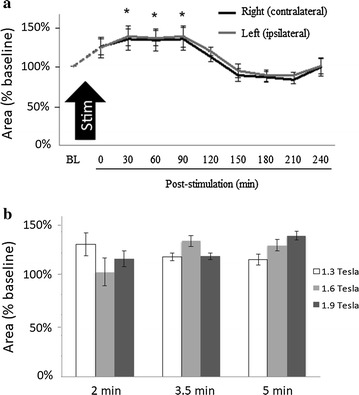
Measures of internal carotid artery dilation after magnetic facial nerve stimulation in pigs. **a** Cross-section area of the internal carotid arteries normalized to baseline. For simplicity, only data from experiments with 1.9 Tesla power for 2 min duration are shown. Repeated measures ANOVA demonstrated no difference between the right- and left-sided measures, but a significant effect between baseline (BL) and post-stimulation (*P < 0.05 versus baseline, paired t-test corrected by the Bonferroni method for multiple comparisons). **b** Change in cross-section area of the ipsilateral internal carotid arteries in relation to stimulation power and stimulation duration. ANOVA showed no significant difference across the various combinations of power and duration (P > 0.40). Mean ± SE; n = 3

Figure 6a shows the increase of cross-section area of the internal carotid arteries normalized to the pre-stimulation baseline area caused by a single stimulation with 1.9 Tesla power for 2 min. The bilateral angiographic measures of the internal carotid artery increased after stimulation according to repeated measure ANOVA comparisons against baseline measures, and significant effects were observed between baseline and 30–90 min post-stimulation. Figure 6b shows the change in cross-section area of the internal carotid artery ipsilateral to facial nerve stimulation across all combinations of stimulation power and stimulation duration. As with the CBF measures (see Fig. 4), ANOVA did not distinguish between the groups.

**Fig. 7:**
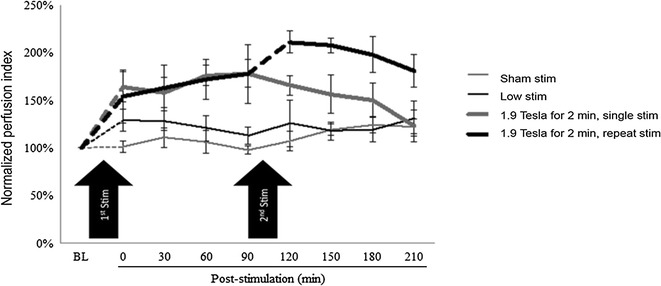
Comparison of a single versus repeated magnetic facial nerve stimulation on cerebral blood flow in pigs. Repeated stimulation at 1.9 Tesla power for 2 min; single stimulation at 1.9 Tesla power for 2 min; 1.0 Tesla power for 1 min (“low stim”); and 0.1 Tesla power for 30 s (“sham stim”). BL, baseline. A paired t test demonstrated a significant effect of single and repeated stimulation at 1.9 Tesla power for 2 min versus sham and low stimulation (P < 0.01). Mean ± SE, n = 3 per group

The dilation of the internal carotid arteries after a single stimulation lasted about 90 min (Fig. 6a). A second stimulation occurring about 100 min after a first stimulation appeared to prolong the elevated CBF response and possibly to potentiate it as well. As shown in Fig. 7, a second stimulation maintained the elevated CBF at a time when the effect of a single stimulation wore-off and CBF began returning toward baseline.

### Study in healthy volunteers

The stimulation parameters for the healthy volunteer study were selected as described in the Methods section. Given the observation that considerably shorter stimulation durations were as effective as 5 min in the pig experiments (see Figs. 4 and 5), we selected a 2-min stimulation period for the healthy volunteer study. As a means to assess tolerability, the stimulation power was increased in a step-wise fashion based on the individual’s willingness to complete a 2-min stimulation period at that power.

Other than the clinical prototype VitalFlow overheating during one stimulation trial, causing it to shut down automatically, no other device events were encountered during the healthy volunteer study. No change in audiometric, ophthalmologic, or neurological examinations was noted in any of the 24 volunteers in Part 1 of the study.

Table 3 shows adverse events reported by the volunteers distributed according to stimulation power received. None of the adverse events persisted after the stimulation was completed, nor was any adverse event judged as serious. No adverse event limited or caused premature termination of the stimulation; all volunteers completed 3 min of stimulation at their selected level of stimulation power.

**Table 3:** Adverse events spontaneously reported by the volunteers and reported in response to the query of the study investigator

N = 35	Maximum stimulation power achieved, Tesla (number of volunteers)					
	< 1.0 T (2)	1.0 T (1)	1.2 T (4)	1.4 T (8)	1.6 T (17)	1.8 T (3)
Expected adverse events during stimulation						
Metallic taste sensation	0	0	1	1	1	0
Vertigo/sensation of movement	0	0	0	1	2	1
Tinnitus/Ringing ears	0	0	0	0	0	0
Adverse events during stimulation						
Visual flashes	0	0	1	2	3	1
Nausea	0	0	0	0	2	1
Sweating	0	0	2	0	7	2
Jaw pain or soreness	0	0	2	5	11	2
Neck pain or soreness	0	0	3	0	1	0
Adverse events after stimulation						
Any	0	0	0	0	0	0

Tolerability is shown by the number of volunteers achieving each level of stimulation power

**Fig. 8:**
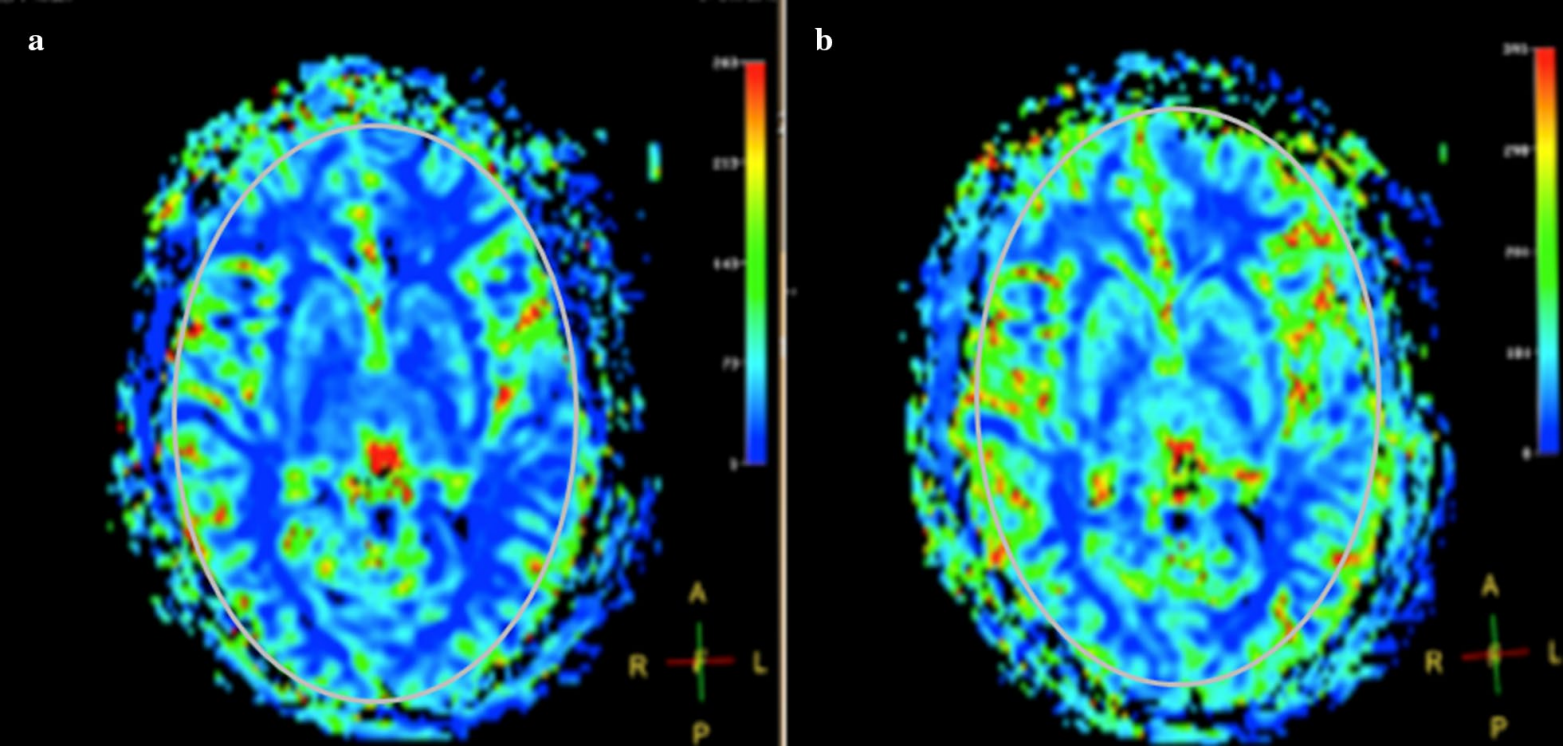
Perfusion index image of cerebral blood flow in a healthy volunteer. **a** Pre-stimulation; **b** post-stimulation. Grey oval shows the used ROI. Volunteer #10, stimulation power = 90%

**Fig. 9:**
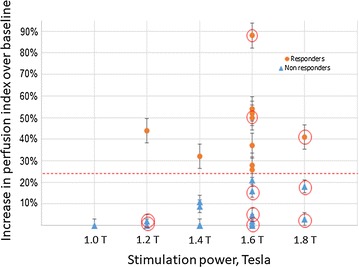
Graph of the responders (≥ 25% change in cerebral blood flow) and the non-responders (< 25% change in cerebral blood flow) in the healthy volunteer study. Ten volunteers were classified as responders and 21 volunteers were classified as non-responders. Ten volunteers exhibited sweating as an adverse event (red circles). Correlation factor is 0.03 ± 0.01

Figure 8 shows the perfusion images of CBF before and after stimulation in a representative volunteer from the responder group (i.e., ≥ 25% increase in CBF). The gray oval shows the ROI used in order to restrict the perfusion index CBF measure for each slice to the brain. Figure 9 shows perfusion index CBF changes over baseline from individual volunteers based on a response ≥ 25% over baseline (“responder”) and a response < 25% (“non-responder”). Overall, 10 volunteers were classified as responders and 21 volunteers were classified as non-responders. No volunteer exhibited a decrease in perfusion index CBF measures after stimulation.

## Discussion

Our aim was to refine the parameters for magnetic stimulation of the facial nerve to increase CBF. As previously published [19], experiments in normal sheep and dog demonstrated CBF increases at a stimulation of 1.5 Tesla power but not at 1.0 Tesla power. Five minutes of stimulation was used in those experiments assuming that it was supramaximal and thus would not fail to induce a CBF response. Here, using the same magnetic stimulation equipment in pigs, we were able to increase CBF with lower stimulation powers and shorter stimulation durations to a degree comparable to what was achieved with higher powers and longer durations of stimulation. Similarly, the pig experiments reported an average CBF increase of 77% over pre-stimulation baseline. Most combinations of stimulation duration and power achieved this level of CBF response in the normal pig, suggesting a steep dose–response curve that rapidly plateaus.

The response to stimulation in our experiments was generally prolonged, lasting 90 min, which is comparable to the effect of magnetic facial nerve stimulation in normal animal and in animals with ischemic stroke [19, 20]. We also observed that, by applying a second stimulation approximately 100 min after the first one, the elevated CBF could be maintained for at least 3.5 h. After the second stimulation, CBF again rapidly increased, then began decaying at a comparable rate to what occurred after the first stimulation.

The increase in CBF suggests that magnetic stimulation of the facial nerve dilates the cerebral arteries. In our experiments, we examined the angiographic response of the internal carotid artery as representative of the cerebral arteries. We observed dilation of the internal carotid arteries on MR angiography that lasted about 90 min following a single stimulation. The MRI techniques used to measure CBF in these experiments thus showed mutually consistent responses and comparable time courses. We also confirm that unilateral stimulation produces bilateral effects on the cerebral vasculature [20].

The pig study may have been biased by the involvement of study team in the delivery of the stimulation and the analysis of the outcome data. However, stimulation parameters were allocated in a random fashion and data analysis was performed through automated processes, which should reduce the bias. Other limitations of this study include the testing of restricted stimulation parameters. This is a necessary limitation given the large number of potential combinations of stimulation factors, including not only stimulation power and duration, but other factors such as pulse shape, pulse duration, and stimulation patterns. Clearly all combinations of these parameters cannot be tested in the preclinical setting, and so we focused on those parameters that are most meaningful to the development of a medical device. Reducing stimulation power and duration directly impacts the design of a medical device since they determine the device’s power demands, electronic tolerances, and cooling requirements. What is more, lower stimulation power and duration can only improve a medical device’s safety and tolerability. Thus, it was the intention of this study to determine how little stimulation power and duration could still effectively increase CBF. Indeed, our initial range of stimulation powers and durations proved to be comparably effective at increasing CBF (see Fig. 4), and we had to add-on additional experiments to define the lower end of a dose–response curve (as per Fig. 5).

Our choice of the pig as a translational animal model reflects the general similarities between the pig head anatomy and human head anatomy [22], which are more closely related than most other, non-primate species. However, the relationship of the pig and human in terms of CBF is less assured, given the connections between the extracranial-intracranial circulation [23], the presence of a carotid rete in the pig, and the quadruped body positioning of the pig. Yet these limitations are generally shared by most of the large animal models available for this research, and so are unavoidable. While interspecies differences do appear to occur, we do not believe additional preclinical testing needs to be done to extrapolate the current findings about pulsed magnetic facial nerve stimulation (e.g., into primates) before advancing the VitalFlow technology into clinical testing.

The pig study showed that we could reduce VitalFlow stimulation power and duration significantly in comparison with our early animal studies. We also recognized the need for tolerability of VitalFlow stimulation in clinical testing: unlike the pigs and other animal experiments, wherein stimulation was always administered under general anesthesia, clinical use of the VitalFlow would be done in awake people. We realized the need to allow people some control over the stimulation power but also the need to achieve the minimum stimulation necessary to increase CBF. Thus, we decided to rapidly increase the stimulation power with the consent of the volunteer over a period of a minute, with the aim of delivering 2 min of stimulation at a power of at least 1.2 Tesla.

This strategy appeared to be successful: with gentle encouragement, volunteers readily could tolerate VitalFlow stimulation for 2 min at 1.2 Tesla or higher power. At stimulation powers greater than 1.2 Tesla, adverse events were encountered. Overall, the clinical prototype VitalFlow demonstrates a better-than-expected adverse event profile, with few adverse events of interest being reported. Common minor adverse events included jaw pain or soreness, sweating on the neck and face, visual flashes, neck pain or soreness, and nausea. None of these adverse events were limiting of stimulation or posed a serious health risk.

Clear responders to stimulation (i.e., a CBF increase of ≥ 25%) represents about a third of all volunteers, and in that group the response to stimulation could be quite sizable. No clear dose–response relationship could be observed in the available dataset, similar to the pig study. Other studies of facial nerve stimulation have reported inconsistent response to facial nerve stimulation [24], with some evidence of intra-animal variability in response to repeated stimulation. In part, this may reflect opposing neural reflex mechanisms and/or arterial autoregulation that serve to maintain a steady level of CBF in normal animals. Indeed, in our healthy volunteer study, we unexpectedly observed a high rate of sweating in the head and neck as an adverse event. Sweating reflects activation of the sympathetic nervous system, which may also counteract a CBF response through vasoconstrictive innervation of the cerebral arteries [25]. Further investigation is needed to understand this intersubject (and potentially intrasubject) variability, and whether or not it occurs in conditions where an increase in CBF is clearly needed by a person, e.g., as in ischemic stroke. We also intend to compare the effectiveness of bilateral versus unilateral stimulation and the ability of repetitive stimulation to maintain elevated CBF in future healthy volunteer studies.

## Conclusions

Magnetic facial nerve stimulation increases CBF using stimulation powers and durations considerably less than what we previously used in our preclinical studies. In human, magnetic facial nerve stimulation was safe, tolerable, and effective at increasing CBF. An unexplained intersubject and intrasubject variability in the response to VitalFlow stimulation was observed in both pigs and man, in agreement with previous research.
